# Correction: Anup C. Katheria. Neonatal Resuscitation with an Intact Cord: Current and Ongoing Trials. *Children* 2019, *6*, 60

**DOI:** 10.3390/children6050071

**Published:** 2019-05-21

**Authors:** Anup C. Katheria

**Affiliations:** 1 Sharp Mary Birch Hospital for Women & Newborns, San Diego, CA 92123, USA; anup.katheria@sharp.com; 2 School of Medicine, Loma Linda University, Loma Linda, CA 92350, USA

The author wishes to make the following corrections to this paper [1]:

The author has made an error in the parameters of the row “PCI-Trial” in Table 2. Table 2, and the paragraph describing Table 2, need to be corrected.

Accordingly, (1)

**Table 2:** Ongoing or planned trials of neonatal resuscitation.

Study	Proposed N	GA (weeks)	Intervention	Time of Cord Clamping, Control Arm	Time of Cord Clamping Intervention Arm	Primary Outcome
VentFirst	940	23–28	CPAP 30–120 s	30–60 s	120 s	IVH
Nep-Cord 3	231	37–41	Resuscitation if needed	<60 s	180 s	SpO2, HR, and Apgar scores in first 10 min
Baby DUCC	120	32–41	Resuscitation if needed	Immediate cord clamping (duration not specified)	Until 1 minute after CO2 detector change or 5 min	Heart Rate at 60 and 120 s
ABC2	660	24–31	Resuscitation if needed	30–60 s	Until stable (approx. 4 min)	Intact Survival (survival without grade 2 ivh or nec)
Nevill and Meyers	120	23–31	Start CPAP and or PPV at 15 s until 60 s	60 s	60 s	Need for blood transfusion
PCI-Trial	202	23–31	Resuscitation if needed	30–60 s	3 min	Composite outcome of severe IVH, BPD, and death

should be replaced with:

**Table 2:** Ongoing or planned trials of neonatal resuscitation.

Study	Proposed N	GA (weeks)	Intervention	Time of Cord Clamping, Control Arm	Time of Cord Clamping Intervention Arm	Primary Outcome
VentFirst	940	23–28	CPAP 30–120 s	30–60 s	120 s	IVH
Nep-Cord 3	231	37–41	Resuscitation if needed	<60 s	180 s	SpO2, HR, and Apgar scores in first 10 min
Baby DUCC	120	32–41	Resuscitation if needed	Immediate cord clamping (duration not specified)	Until 1 minute after CO2 detector change or 5 min	Heart Rate at 60 and 120 s
ABC2	660	24–31	Resuscitation if needed	30–60 s	Until stable (approx. 4 min)	Intact Survival (survival without grade 2 ivh or nec)
Nevill and Meyers	120	23–31	Start CPAP and or PPV at 15 s until 60 s	60 s	60 s	Need for blood transfusion
PCI-Trial	202	23–29	Resuscitation if needed	Intact cord milking × 4	3 min	composite outcome of severe IVH, chronic lung disease or death

(2) Lastly, Presti et al. are proposing a multicenter trial (PCI-Trial, NCT02671305) comparing intact umbilical cord milking to delayed cord clamping for 3 min with assistance when needed in premature infants <32 weeks. Their primary outcome is a composite outcome of severe IVH, BPD, and death with a sample size of 202 infants.

should be replaced with:

Lastly, Pratesi et al. are proposing a multicenter trial (PCI-Trial, NCT02671305) comparing intact umbilical cord milking to delayed cord clamping for 3 min with assistance when needed in premature infants <30 weeks. Their primary outcome is a composite outcome of severe IVH, BPD, and death with a sample size of 202 infants.

(3) Presti et al. completed a pilot feasibility study comparing 3 min delay with resuscitation when needed to intact umbilical cord milking four times. Infants with delayed cord clamping had a higher 5 min Apgar, but a lower admission temperature [16].

should be replaced with:

Pratesi et al. completed a pilot feasibility study comparing 3 min delay with resuscitation when needed to intact umbilical cord milking four times. Infants with delayed cord clamping had a higher 5 min Apgar, but a lower admission temperature [16].

The author apologizes for any inconvenience caused to the readers by these changes.
